# Is music intervention effective in reducing anxiety and pain during breast biopsy procedure? A systematic review and meta-analysis of randomized controlled trials

**DOI:** 10.1007/s00520-022-07414-7

**Published:** 2022-11-09

**Authors:** Ahmed S. A. Ashour, Mohamed Abd-ElGawad, Mariam Yohanna, Mostafa El-Nagar, Ahmed Nasser Fadl, Gehad Mohammed Goda, Yassamine Ouerdane, Hany Saad, Mona Fouad, Noura El-Nassery, Mohamed Abdelmonem Kamel, Iman Ezahaby

**Affiliations:** 1 Department of Obstetrics and Gynecology, Faculty of Medicine, Cairo University, Cairo, Egypt; 2 Faculty of Medicine, Fayoum University, Fayoum, Egypt; 3 Faculty of Medicine, Saad Dahleb University, Blida, Algeria

**Keywords:** Anxiety, Breast biopsy, Music, Pain relief, Symphony

## Abstract

**Purpose:**

To evaluate the evidence from randomized clinical trials (RCTs) about the effect of music intervention in reducing patients’ anxiety during breast biopsy.

**Methods:**

Electronic databases including PubMed, Cochrane Library, Scopus, and Web of Science were searched using the relevant MeSH terms. The inclusion criteria were all RCTs assessing the effect of music therapy versus no music in reducing anxiety during breast biopsy. The extracted outcomes were anxiety and pain during breast biopsy. They were pooled as mean difference (MD) with a 95% confidence interval (CI) in a fixed-effects model, using Review Manager 5.3 software for windows. The quality of included studies was assessed with the Cochrane risk of bias assessment tool (RoB 1.0). Then, the outcomes of our meta-analyses were independently evaluated by the Grading of Recommendations Assessment, Development and Evaluation (GRADE) to know the grade of their evidence.

**Results:**

The final analysis included five RCTs. We found a positive effect of music therapy in reducing anxiety levels compared with control group (MD =  − 2.11; 95% CI (− 4.16 to − 0.06); *p* = 0.04). No difference between music and control groups regarding pain associated with breast biopsy (MD = 0.22; 95% CI (− 0.81 to 1.25); *p* = 0.68). The GRADE rating of our outcomes was low for anxiety levels and very low for pain during the biopsy.

**Conclusions:**

Music therapy could be an effective, simple, non-pharmacological option in relieving anxiety during breast biopsy; however, it had no effect on procedure-associated pain. More large and high-quality studies are needed to confirm our results.

## Introduction

A breast biopsy is a procedure in which a sample of breast tissue is extracted and prepared to be sent to further laboratory testing [1]. The most frequent indication for breast biopsy is the suspicion of malignant lesions by screening mammography [2].

Breast cancer is the second most commonly diagnosed cancer worldwide, including low- and middle-income countries [3]. The incidence rates are highest in North America, Australia/New Zealand, and western and northern Europe and lowest in Asia and sub-Saharan Africa [4]. Notably, it became a significant public health issue due to its associated high morbidity, mortality, and massive health costs. Managing the psychological health of women is of extreme importance in all the stages of the disease, starting from the first suspicion to the different treatment modalities. Anxiety, cognitive defense, and emotion-focused coping are usually witnessed among patients with suspected breast cancer awaiting diagnosis [5]. In particular, prior to breast biopsy, patients experience general anxiety and specific worry about both the procedure itself and the biopsy results (i.e., the diagnosis) [6]. Such anticipatory emotional distress has been related to poorer biopsy-related outcomes, including increased pain and physical discomfort [7].

At present, tissue biopsy is a very useful clinical tool that can assist with critical clinical decision-making in patients with suspicious lesions. In the USA, 1.7 million women undergo breast biopsies each year [8]. Breast biopsy methods include core needle biopsy (CNB), fine needle aspiration (FNA), surgical biopsy, and skin punch biopsy [9]. The difference between CNB and FNA is the size of the needle. Both procedures do not require incisions, unlike the open (surgical) biopsy in which a cut is made in the breast. These techniques are usually done with the help of guided tools such as vacuum-assisted or ultrasound-guided biopsies [9, 10].

Although the biopsy is a simple technique that can only induce slight damage, its adverse biopsy-related outcomes cannot be neglected. Breast biopsy patients typically have substantial levels of anticipatory emotional distress [11]. Moreover, a painful feeling after the biopsy is another chief complaint of patients [12]. Several studies reported that pain perception ranges from 2.0 to 5.8 out of 10 during imaging-guided breast biopsy and 3.3 to 4.6 out of 10 for vacuum-assisted (VA) biopsies [13–15]. Therefore, emotional distress and pain have been the most challenging problems of the biopsy, which need to be urgently resolved. Effectively managing the perception of pain is important for the good sake of the patients and could eventually affect patient satisfaction and the practice revenues.

Pain is a subjective and complex phenomenon, and its perception could be influenced by genetic, developmental, familial, psychological, social, and cultural variables [16]. Several pharmacological and non-pharmacological interventions could help minimize the sensation of pain, including listening to music [17]. Music therapy is an easily accessible and inexpensive method used by many people to regulate moods and emotions in their daily lives [18]. To date, music interventions have been widely used in health care. Cochrane reviews have demonstrated beneficial effects of listening to music on anxiety in patients with cancer, coronary heart disease, or preoperative anxiety [19–21].

There is controversy regarding the role of music in patients undergoing breast biopsy. While music had promising results in reducing pain and anxiety during breast biopsy [22], other recent RCTs concluded that music intervention has no impact on pain perception [23, 24]. Therefore, we aimed to synthesize evidence on the role of music therapy in reducing anxiety and pain of patients undergoing breast biopsy.

## Methods

### Study design

This is a systematic review and meta-analysis study for all RCTs showing the effect of music on reducing pain and anxiety in women undergoing breast biopsy. We followed the Preferred Reporting Items for Systematic Reviews and Meta-Analyses (PRISMA) guidelines in this study [25]. Because the study was a systematic review, it was exempt from ethics approval and obligate protocol registration. Therefore, the study’s protocol was not online registered.

### Search strategy

We performed a comprehensive and systematic search of four electronic databases, PubMed, Cochrane CENTRAL Library, Scopus, and Web of Science, from their inception till September 2021, without language restrictions. The following keywords were used: ((Breast OR breasts) AND (biopsy OR “Fine needle aspiration” OR FNA OR “Core needle biopsy”)) AND (pain OR pains OR Suffering OR Ache OR Aches OR Anxiety OR Angst OR Nervousness OR Hypervigilance OR Anxiousness OR Anxieties) AND (music OR Symphony OR Rhythm OR Orchestra OR Song OR (Vocal AND (Melodies OR Melody))). In addition, we searched the references of relevant articles for potentially relevant studies.

### Eligibility criteria

The inclusion criteria were as follows: (1) studies including women undergoing breast biopsy; (2) studies in which music interventions such as music, song, or any equivalent were applied during breast biopsy; (3) studies including a control group without music interventions; (4) studies reporting anxiety and pain as their primary outcome; and (5) RCTs. We excluded from our study all non-English studies, non-randomized clinical trials, editorials and animal studies, case reports, case series, notes, letters, posters, thesis, books, conference proceedings. We excluded any paper not reporting enough data to report our included outcomes.

### Outcomes

Anxiety and pain outcomes as evaluated by the State anxiety scale (SAS) and visual analogue scale (VAS), respectively. The anxiety change score is the difference in pre-biopsy and post-biopsy anxiety levels.

### Study selection and data extraction

We followed a two-stage process for screening and selection of eligible studies after the removal of duplicates. Two reviewers independently screened the titles and abstracts of retrieved search records that agree with the selection criteria in the first stage. In the second stage, the full texts of potentially eligible studies were screened for a final decision about eligibility. If a disagreement occurred, another author was included in the discussion to achieve consensus.

Three authors independently reviewed each included article and extracted the following data using a standardized Microsoft Excel sheet: first author, year of publication, number of enrolled participants, characteristics of participants, details of intervention, and main findings (Table 1). In case of incomplete data, we contacted the original study’s authors to request the missing details.

**Table 1:** General and baseline characteristics

Study ID	Study design	Groups of interventions	Sample size	Age (mean ± SD)	Race	Education	Marital status	Breast biopsy type	Conclusion
Akın 2021	Randomized controlled trial	Music	31	44.2 ± 11.0	*	20 (64.5%) participants received < 12 years of education	*	Ultrasound-guided core needle breast biopsy	Music reduced anxiety, but not pain compared to the standard
		Standard	33	43.1 ± 12.4	*	20 (60.6%) participants received < 12 years of education	*		
Bennett et al. 2020	Randomized controlled trial	Music	75	50.7 ± 10.5	62 white and 13 not white	32 graduate, 20 college, and 23 less than college	*	Stereotactic or ultrasound-guided core biopsy	Music reduced anxiety compared to the standard
		Standard	54	48.4 ± 14.25	44 white and 10 not white	19 graduate, 18 college, and 17 less than college	*		
Bugbee et al. 2005	Randomized controlled trial	Medication	48	53 ± 10	38 white and 10 not white	12 graduate, 26 college, and 10 less than college	35 married, 10 divorced, 2 single, and 1 not specified	Core needle biopsy	Oral anxiolytic medication before breast biopsy reduces anxiety
		Music	47	57.4 ± 10.25	39 white and 8 not white	12 graduate, 23 college, and 10 less than college	35 married, 2 divorced, 6 single, and 4 not specified		
		Standard	48	53.2 ± 11.5	40 white and 8 not white	10 graduate, 28 college, and 9 less than college	31 married, 7 divorced, 6 single, and 4 not specified		
Soo et al. 2016	Randomized controlled trial	Loving-kindness meditation	41	56.10 ± 13.04	34 white and 7 black	15.23 ± 3.29 years of education	25 married	Stereotactic or ultrasound-guided core biopsy	Music reduced anxiety, but not pain compared to the standard
		Music	40	52.93 ± 11.08	24 white and 16 black	15.55 ± 3.59 years of education	21 married		
		Standard	40	49.85 ± 12.78	27 white, 9 black, and 4 Asian	15.27 ± 4.28 years of education	21 married		
Wren et al. 2019	Randomized controlled trial	Loving-kindness meditation	23	57.61 ± 11.87	19 white, 3 African, and 2 Asian	15.62 ± 3.02 years of education	12 married	Stereotactic or ultrasound-guided core biopsy	Music reduced pain, but not anxiety compared to the standard
		Music	16	57.31 ± 7.53	11 white and 4 African	15.93 ± 2.34 years of education	10 married		
		Standard	17	52.35 ± 13.03	9 white and 5 African	15.27 ± 3.75 years of education	10 married		

### Quality assessment

We assessed the risk of bias using the Cochrane risk of bias assessment tool (RoB 1.0) described in the Cochrane Handbook for Systematic Reviews of Interventions [26]. Seven domains were evaluated: random sequence generation (selection bias), allocation sequence concealment (selection bias), blinding of participants and personnel (performance bias), blinding of outcome assessment (detection bias), incomplete outcome data (attrition bias), selective outcome reporting (reporting bias), and other potential sources of bias. Each domain was judged as having a low, high, or unclear risk of bias. All assessments were independently fulfilled by the same two authors and discrepancies were resolved through discussion. Then, the outcomes of our meta-analyses were independently evaluated by the Grading of Recommendations Assessment, Development and Evaluation (GRADE) to know their grade of the evidence [27].

### Publication bias

According to Egger and colleagues, the estimation of publication bias utilizing funnel plot and Egger’s test is unpredictable for less than ten pooled studies. Therefore, in the current study, we could not assess for publication bias by Egger’s test due to the limited number of the studies which met our inclusion criteria [28].

### Statistical analysis

Continuous outcomes were pooled as weighted mean difference (WMD) using the Mantel-Hansel method with 95% confidence intervals (CI). Review Manager 5.3 software (Cochrane Information Management System) was used to perform the calculations. Heterogeneity was evaluated graphically using forest plots and statistically using the Cochrane *Q* test and *I*^2^ statistics [29]. In the *Q*-tests, *p* < 0.1 and *I*^2^ ≥ 50% were considered indicative of statistically significant heterogeneity. Statistically significant results were considered when the *p*-value was < 0.05.

If there was any heterogeneity between studies, we used the random-effects model; otherwise, the fixed-effects model was utilized. We did the sensitivity analysis to consider the contribution of each included study to the pooled estimation of the reported heterogeneity by removing one trial at a time and reanalyzing the pooled mean difference estimation for the remaining studies.

## Results

### Search results and characteristics of included studies

**Fig. 1:**
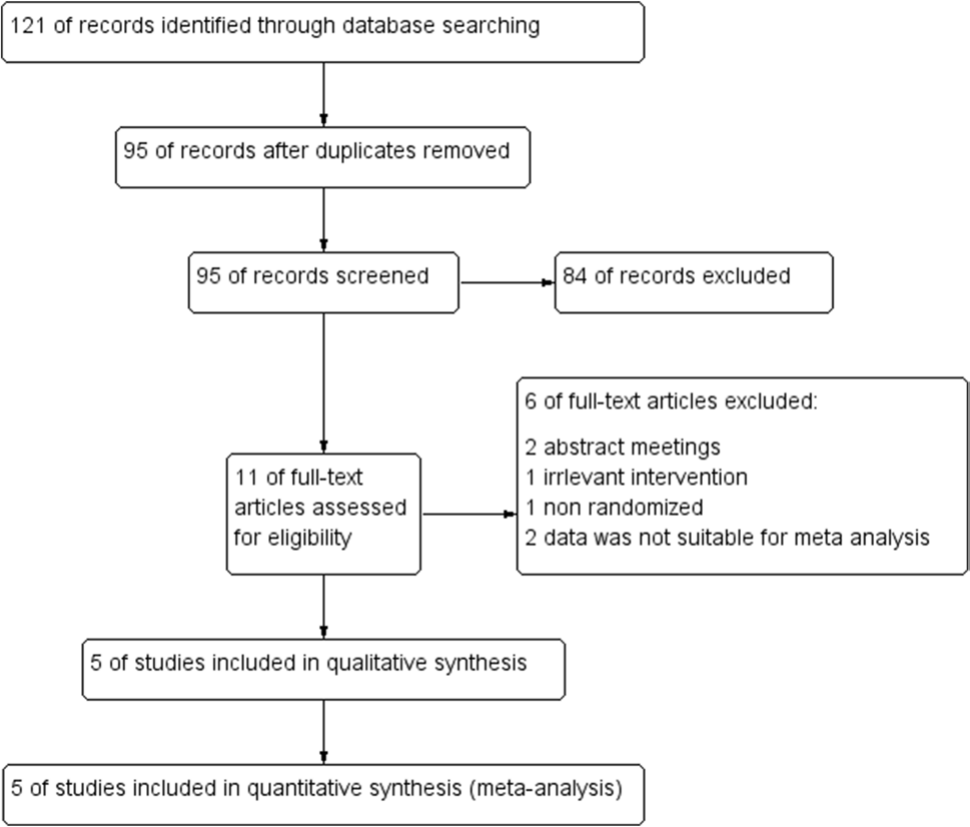
Study flow diagram (PRISMA)

Initially, the search results yielded 121 records. After removing the duplicates, we performed title and abstract screening for 95 studies; only 11 were eligible for full-text screening. Of them, five RCTs (401 patients) were included in the meta-analysis as shown in the PRISMA flow diagram (Fig. 1). The baseline characteristics of patients and a summary of included studies are shown in Table 1.

### Risk of bias assessment

**Fig. 2:**
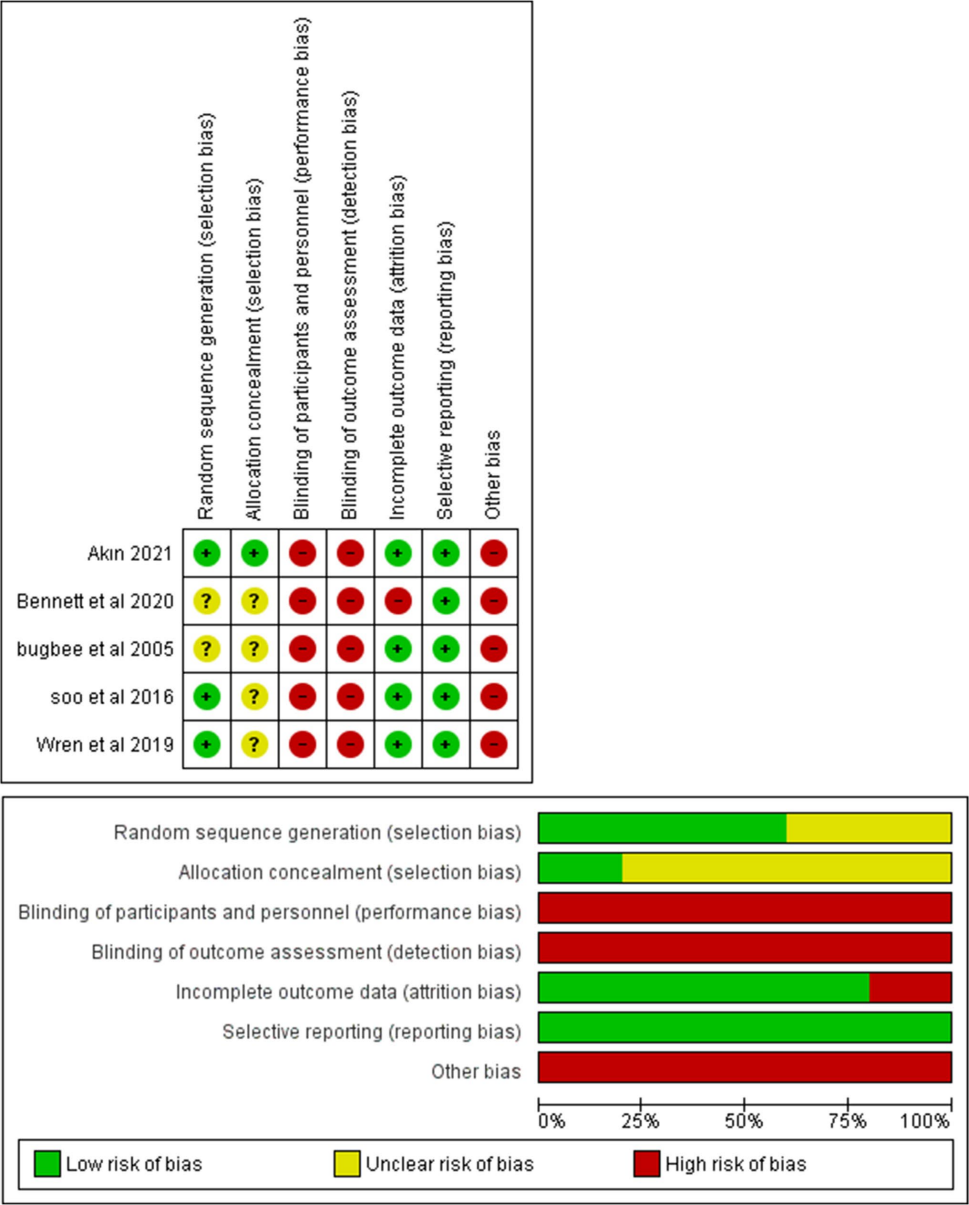
Risk of bias summary of the included studies

Using the risk of bias tool of Cochrane described in Cochrane Handbook for Systematic Reviews of Interventions, we found that the quality of included studies was unclear in selection bias because most of the included studies did not mention the methods of randomization or allocation concealment. There was low quality regarding the performance and detection bias criteria because it was not possible to blind participants, physicians, and the outcomes assessors due to the nature of intervention. There was no attrition or reporting bias except for Benett et al. [30] study which had high risk of bias as there were no data for some patients who had the procedure performed. We considered all included studies as having high risk of other sources of bias due to unavailability of their protocols. The summary of quality assessment domains of included studies is shown in Fig. 2.

### Outcomes

1. State anxiety score
  Pooled data from five studies [22–24, 30, 31] (*N* = 401 patients) showed a lower anxiety score in music group compared with control group (no music) (MD =  − 2.11; 95% CI (− 4.16 to − 0.06); *p* = 0.04; Fig. 3). Pooled studies were homogenous (*p* = 0.35).

**Fig. 3:**
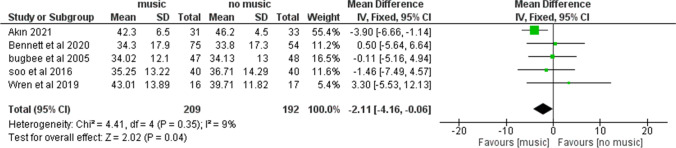
State anxiety score comparing a group of patients who listened to music and another group who did not listen to music during a breast biopsy
2. Anxiety decrease
  Anxiety decrease was reported in four studies (178 participants in music group and 159 in control group). The overall effect showed that music significantly reduced anxiety level compared to control (MD = 3.37; 95% CI (0.17 to 6.57); *p* = 0.04; Fig. 4). Pooled studies were homogenous (*p* = 0.84).

**Fig. 4:**
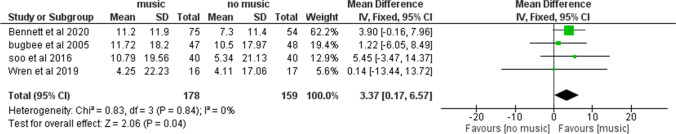
Decrease in state anxiety score comparing a group of patients who listened to music and another group who did not listen to music during a breast biopsy
3. Pain during breast biopsy

**Fig. 5:**
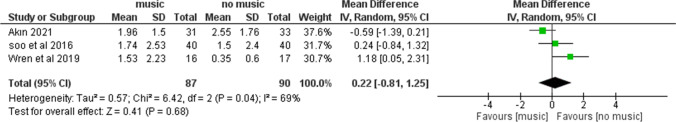
Visual analogue scale score for pain comparing a group of patients who listened to music and another group who did not listen to music during a breast biopsy

Pain levels during the procedure were reported in three studies (87 participants in music group and 90 participants in control group). The pooled MD showed no significant differences in pain scores between both groups (MD = 0.22; 95% CI (− 0.81 to 1.25); *p* = 0.68; Fig. 5). Pooled studies were heterogeneous under a random effect model (*P* = 0.04; *I*^2^ = 69%).

### Summary of the findings and GRADE evaluation of the outcomes (Table 2)

**Table 2:** Summary table, GRADE rating

Outcome name	Number of included studies	Design of included studies	Mean difference, 95% CI	Heterogeneity	Number of patients in Music group	Number of patients in control	Risk of bias	Inconsistency	Indirectness	Imprecision	Other considerations^a^	Quality
State anxiety score	Five studies with 401 patients	RCTs	− 2.11, (− 4.16, − 0.06)	*I*^2^ = 9%, P = 0.35	209	192	Serious^b^	Not serious	Not serious	Serious^d^	Not existed	Low ⊕ ⊕ ◯◯
Decrease in state anxiety score	Four studies with 337 patients	RCTs	3.37, (− 0.17, 6.57)	*I*^2^ = 0%, P = 0.84	178	159	Serious^b^	Not serious	Not serious	Serious^d^	Not existed	Low ⊕ ⊕ ◯◯
Pain during breast biopsy	Three studies with 177 patients	RCTs	0.22, (− 0.81, 1.25)	*I*^2^ = 69%, P = 0.04	87	90	Serious^b^	Serious^c^	Not serious	Serious^d^	Not existed	Very low ⊕ ◯◯◯

RCTs, randomized control trials; CI, confidence interval

^a^Other considerations are publication bias, large effect, dose response, and plausible confounding factors

^b^As the included studies showed higher risk of bias especially with blinding of participants, personnel, and outcome assessors besides other bias

^c^As the outcome had significant heterogeneity

^d^As the analysis included small number of patients with wide confidence interval

Low indicates that the confidence about the result is limited and the true effect can be different from our result

Very low indicates that confidence about the result is very little and the true effect is more probably to be different from out result

All outcomes were evaluated by GRADE criteria. The state anxiety score and decrease in anxiety score showed low quality which indicated that the confidence about the results was limited and the difference between the actual effect and our result could exist. This was because of the increased risk of bias in the included studies and imprecision. In the pain during the breast biopsy, the GRADE rating was very low which indicated that the confidence about the results was very little and the difference between the actual effect and our result was more probably to present. This was because of the increased risk of bias in included studies, imprecision, and heterogeneity.

## Discussion

### Summary of the results

To our knowledge, there are no other systematic reviews or meta-analyses of RCTs that investigated music intervention for reducing anxiety and pain caused by the biopsy. However, several RCTs with different results have been reported. For this meta-analysis, data across all the included studies were evaluated to verify the role of music intervention with more precision than any single study. Our meta-analysis showed that Music intervention is readily effective in reducing anxiety during the breast biopsy. However, there is no significant difference between both groups regarding pain during the breast biopsy procedure.

### Significance of the results

The experience of anxiety before and during the insertion of the biopsy needle is particularly common. Dealing with the fear of needles and anxiety is important to control pain perception since lower levels of anxiety, and anticipated pain is closely related to lower levels of procedure-related pain [32]. Various factors could potentially affect and mediate the perception of stressful events, including coping style, social support, personality type, and gender [33].

For many reasons, non-pharmacological interventions have gained considerable attention in reducing pain and anxiety due to the absence of side effects, lower costs, and ease and comfortable atmosphere they create. Remarkable results have been reported on the use of music intervention as a modality to reduce anxiety. Paskin and Baker’s works showed that relaxing music reduces subjective anxiety in patients compared to those who waited in silence in the surgery waiting room [34]. Several RCTs yielded similar results when applying this intervention during the biopsy procedure, and our results agreed with those deduced from different interventional procedures [35, 36]. Additionally, music intervention showed a potential impact in reducing anxiety during a breast biopsy, as described in our meta-analysis.

### Explanation of the results

The anxiolytic effect of music might be due to the modulation of nervous and endocrine systems and its effect on neurotransmitters, hormones, cytokines, and immunoglobulins, as well as a psychological response [37]. Listening to music may suppress the sympathetic system, decreasing the cortisol levels and triggering activity in brain regions linked to emotional experiences and modulating anxiety levels [38]. Music leads to distraction which may mitigate pain by modulating connectivity between pain centers in the brain [39] or simply by making time pass more quickly while waiting for the vacuum-assisted biopsy to finish [30].

A previous systematic review and meta-analysis by Lin et al. [18] showed that music interventions during pregnancy may decrease maternal anxiety. A similar review concluded that music was beneficial in reducing anxiety scores and physiological indexes related to anxiety (e.g., heart rate) in pregnant women undergoing cesarean section or labor process [40].

As for pain perception, our analysis showed no significant effect on pain perception during breast biopsy, and the result is comparable with other meta-analyses performed on biopsies from different body regions [41, 42] despite without controversy [43]. Our results clearly show that pain was not affected with music intervention in breast biopsy. Our results agreed with the results of Abdelhakim et al. [44] systematic review and meta-analysis which concluded that music therapy was ineffective in lowering pain scores during colposcopy procedures.

A recent meta-analysis by Song et al. [36] aimed to evaluate the efficacy of music therapy for reducing the anxiety and pain of patients who underwent a biopsy. Nine RCTs were included. Music was successful in decreasing systolic blood pressure before the biopsy, State-Trait Anxiety Inventory scores after the biopsy, diastolic blood pressure after the biopsy, and heart rate after the biopsy. Similarly, music also tended to be more effective for controlling pain after the biopsy.

It is important to note that individual music preference is important to the effect of a music intervention [30] which was not the case in most of our included RCTs since the music was already chosen by the experimenters [23, 31]. Our pooled results from all the included data support the usefulness of listening to music in reducing patient anxiety during a biopsy to soothe and relieve patients’ feelings, compared with the standard care conditions. Unfortunately, there is no clinical significance in reducing pain perception on VAS score. Furthermore, future studies are warranted to establish more objective pain assessment methods and more ideal techniques for RCTs.

### Clinical implications

Music interventions are inexpensive and practical. In addition, they are safer and have no significant side effects compared with pharmacological treatment. Thus, their application in daily care may be advisable for women.

### Strengths of the study

The strength of the present study is that we performed comprehensive search of multiple databases without language restrictions. We included five RCTs in the quantitative analysis constituting a strong evidence level.

### Study limitations

The main limitations of our study are the relatively small sample size in some trials and the deficiency of blinding and allocation concealment in the risk of bias assessment which is attributed to the difficulty to blind the relevant parties. However, the nature of music interventions makes it difficult to apply double-blinded studies. Although we excluded non-English studies, recent evidence realized that excluding non-English studies does not cause any bias to the meta-analysis results [45]. Another limitation is evaluating pain with a VAS score which is not an objective method and can be influenced by several factors, such as social and cultural status. Another limitation was the lack of a good standard of music intervention, such as playing type, timing, and musical style since diverse music interventions can have different physical, neurological, and psychological states. Also, the GRADE rating of our outcomes was low with state anxiety score and decrease in state anxiety score while it was very low with pain during a breast biopsy. This could be explained by the limited published number of studies related to this topic and the nature of the intervention (music) and control (no music) which made it difficult to blind the study’s personnel, participants, and assessors which increased the risk of bias. Therefore, we recommend performing more studies with a larger population and of high quality.

## Conclusion

This systematic review suggests that music therapy has a great positive effect in reducing anxiety levels when compared with control groups during the breast biopsy procedure. However, it has no effect in reducing pain during breast biopsy. The estimate of music intervention effect might be exaggerated because no blinding or allocation concealment was applied in included trials, preventing the analysis of a subjective outcome.
